# *N* ^1^,*N* ^4^-Bis(2-thienylmethyl­ene)cyclo­hexane-1,4-diamine

**DOI:** 10.1107/S1600536809035545

**Published:** 2009-09-09

**Authors:** Kyung-Eun Lee, Soon W. Lee

**Affiliations:** a Department of Chemistry (BK21), Sungkyunkwan University, Natural Science Campus, Suwon 440-746, Republic of Korea

## Abstract

The title compound, C_16_H_18_N_2_S_2_, lies about an inversion center with only half of the mol­ecule in the asymmetric unit. The cyclo­hexane ring adopts a chair conformation, and the terminal thio­phene rings are in a *transoid* orientation, with an S⋯S separation between the two terminal 2-thio­phene rings of 11.6733 (9) Å.

## Related literature

For a general introduction to coordination polymers, see: Batten *et al.* (2009 ▶); Perry *et al.* (2009 ▶); Robin & Fromm (2006 ▶). For structurally related compounds, see: Yun *et al.* (2009 ▶). For related linking ligands containing terminal thio­phene rings, see: Lee & Lee (2007 ▶); Huh *et al.* (2008 ▶); Kim & Lee (2008 ▶).
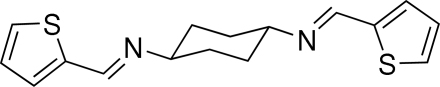


## Experimental

### 

#### Crystal data

C_16_H_18_N_2_S_2_
*M* *_r_* = 302.44
Monoclinic, 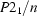

*a* = 6.2173 (4) Å
*b* = 7.4999 (5) Å
*c* = 17.1289 (12) Å
β = 97.047 (3)°
*V* = 792.67 (9) Å^3^
*Z* = 2
Mo *K*α radiation
μ = 0.33 mm^−1^
*T* = 296 K
0.46 × 0.24 × 0.20 mm

#### Data collection

Bruker SMART CCD area-detector diffractometer
Absorption correction: multi-scan (*SADABS*; Sheldrick, 1996 ▶) *T*_min_ = 0.864, *T*_max_ = 0.937
9045 measured reflections
1889 independent reflections
1590 reflections with *I* > 2σ(*I*)
*R*_int_ = 0.022

#### Refinement

*R*[*F* ^2^ > 2σ(*F* ^2^)] = 0.032
*wR*(*F* ^2^) = 0.089
*S* = 1.02
1889 reflections
127 parameters
All H-atom parameters refined
Δρ_max_ = 0.19 e Å^−3^
Δρ_min_ = −0.18 e Å^−3^

### 

Data collection: *SMART* (Bruker, 1997 ▶); cell refinement: *SAINT* (Bruker, 1997 ▶); data reduction: *SAINT*; program(s) used to solve structure: *SHELXS97* (Sheldrick, 2008 ▶); program(s) used to refine structure: *SHELXL97* (Sheldrick, 2008 ▶); molecular graphics: *SHELXTL* (Sheldrick, 2008 ▶); software used to prepare material for publication: *SHELXTL*.

## Supplementary Material

Crystal structure: contains datablocks global, I. DOI: 10.1107/S1600536809035545/pv2205sup1.cif

Structure factors: contains datablocks I. DOI: 10.1107/S1600536809035545/pv2205Isup2.hkl

Additional supplementary materials:  crystallographic information; 3D view; checkCIF report

## Figures and Tables

**Fig. 1.**
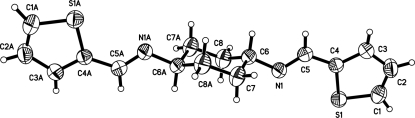
Molecular structure of the title compound showing 50% probability displacement ellipsoids. Symmetry code for the atoms with A in their labels: -x+2, -y+2, -z+2.

## supplementary crystallographic information

### Comment

Design and construction of coordination polymers (or metal–organic frameworks, MOFs) are currently under intensive study due to their desirable applications in catalysis, nonlinear optical activity, spin crossover, luminescence, long-range magnetism, adsorption–desorption, and gas storage (Batten *et al.*, 2009; Perry IV *et al.*, 2009; Robin & Fromm, 2006). In preparing such polymers, appropriate linking ligands play a fundamental role. We have continually reported long bis(pyridine)-, bis(furan)-, bis(thiophene)-, and (pyridine–amine)-type linking ligands and their coordination polymers (Yun *et al.* 2009). As an extension to our ongoing study of novel linking ligands and their coordination polymers, we have prepared a long, potential linking ligand containing an intervening cyclohexane ring with two terminal thiophene rings.

The molecular structure of the title compound (Fig. 1) contains an intervening cyclohexane ring between two iminethiophene (—*N*═CH—2-thiophene) fragments. The cyclohexane ring fragment adopts a chair conformation. The imine fragments occupy the equatorial sites of the cyclohexane ring and are *trans* with respect to each other. The terminal thiophene rings also adopt an overall *transoid* conformation. The S···S separation between the two terminal 2-thiophene rings is 11.6733 (9) Å. Several related linking ligands containing terminal thiophene rings were previously employed to obtain coordination networks: (2-thiophene)—CH═*N*—*N*═CH—(2-thiophene) (Lee & Lee, 2007; Huh *et al.*, 2008) and (3-thiophene)—CH═*N*—*N*═CH—(3-thiophene) (Kim & Lee, 2008).

### Experimental

At room temperature, *trans*-1,4-diaminocyclohexane (1.0 g, 8.76 mmol) was added to 2-thiophene carboxaldehyde (1.72 ml, 18.72 mmol) in 80 ml methanol. After adding dichloromethane (50 ml) and three drops of formic acid, the mixture was stirred for 15 h, and then the solvent was removed under vacuum. The resulting solid was extracted with dichloromethane (150 ml) and washed with water (30 ml × 3). The organic phase was dried over MgSO_4_ and then filtered. All the solvent was removed to give white crude solid, which was recrystallized from dichloromethane/hexane to give colorless crystals of the title compound suitable for X-ray crystallographic study (2.05 g, 6.78 mmol, 77%). mp: 511–513 K.

### Refinement

All H atoms were located from difference maps and refined isotropically.

### Crystal data

**Table:**

C_16_H_18_N_2_S_2_	*F*(000) = 320
*M**_r_* = 302.44	*D*_x_ = 1.267 Mg m^−^^3^
Monoclinic, *P*2_1_/*n*	Mo *K*α radiation, λ = 0.71073 Å
Hall symbol: -P 2yn	Cell parameters from 5365 reflections
*a* = 6.2173 (4) Å	θ = 2.4–28.2°
*b* = 7.4999 (5) Å	µ = 0.33 mm^−^^1^
*c* = 17.1289 (12) Å	*T* = 296 K
β = 97.047 (3)°	Block, colourless
*V* = 792.67 (9) Å^3^	0.46 × 0.24 × 0.20 mm
*Z* = 2	

### Data collection

**Table:**

Bruker SMART CCD area-detector diffractometer	1889 independent reflections
Radiation source: sealed tube	1590 reflections with *I* > 2σ(*I*)
graphite	*R*_int_ = 0.022
φ and ω scans	θ_max_ = 28.3°, θ_min_ = 2.4°
Absorption correction: multi-scan (*SADABS*; Sheldrick, 1996)	*h* = −8→8
*T*_min_ = 0.864, *T*_max_ = 0.937	*k* = −8→9
9045 measured reflections	*l* = −22→19

### Refinement

**Table:**

Refinement on *F*^2^	Primary atom site location: structure-invariant direct methods
Least-squares matrix: full	Secondary atom site location: difference Fourier map
*R*[*F*^2^ > 2σ(*F*^2^)] = 0.032	Hydrogen site location: inferred from neighbouring sites
*wR*(*F*^2^) = 0.089	All H-atom parameters refined
*S* = 1.02	*w* = 1/[σ^2^(*F*_o_^2^) + (0.0484*P*)^2^ + 0.1406*P*] where *P* = (*F*_o_^2^ + 2*F*_c_^2^)/3
1889 reflections	(Δ/σ)_max_ = 0.001
127 parameters	Δρ_max_ = 0.19 e Å^−^^3^
0 restraints	Δρ_min_ = −0.18 e Å^−^^3^

### Special details

**Table:**

Experimental. IR (KBr, cm^-1^): 3443 (w), 3103 (w), 2923 (*m*), 2852 (w), 1626 (*s*), 1430 (*m*), 1306 (w), 1210 (*m*), 1084 (*m*), 944 (*m*), 847 (*m*), 730 (*s*), 498 (*m*).
Geometry. All e.s.d.'s (except the e.s.d. in the dihedral angle between two l.s. planes) are estimated using the full covariance matrix. The cell e.s.d.'s are taken into account individually in the estimation of e.s.d.'s in distances, angles and torsion angles; correlations between e.s.d.'s in cell parameters are only used when they are defined by crystal symmetry. An approximate (isotropic) treatment of cell e.s.d.'s is used for estimating e.s.d.'s involving l.s. planes.
Refinement. Refinement of *F*^2^ against ALL reflections. The weighted *R*-factor *wR* and goodness of fit *S* are based on *F*^2^, conventional *R*-factors *R* are based on *F*, with *F* set to zero for negative *F*^2^. The threshold expression of *F*^2^ > σ(*F*^2^) is used only for calculating *R*-factors(gt) *etc*. and is not relevant to the choice of reflections for refinement. *R*-factors based on *F*^2^ are statistically about twice as large as those based on *F*, and *R*- factors based on ALL data will be even larger.

### Fractional atomic coordinates and isotropic or equivalent isotropic displacement parameters (Å^2^)

**Table:**

	*x*	*y*	*z*	*U*_iso_*/*U*_eq_	
S1	0.65706 (5)	0.42717 (5)	0.78995 (2)	0.04947 (14)	
N1	0.78346 (18)	0.71526 (15)	0.91441 (7)	0.0458 (3)	
C1	0.4495 (3)	0.2897 (2)	0.75469 (10)	0.0554 (4)	
C2	0.2791 (3)	0.3032 (2)	0.79591 (10)	0.0575 (4)	
C3	0.3138 (2)	0.42688 (19)	0.85814 (9)	0.0488 (3)	
C4	0.51420 (19)	0.50601 (18)	0.86246 (7)	0.0396 (3)	
C5	0.6024 (2)	0.64140 (18)	0.91770 (7)	0.0412 (3)	
C6	0.8511 (2)	0.85271 (18)	0.97279 (8)	0.0430 (3)	
C7	0.8308 (3)	1.0366 (2)	0.93470 (10)	0.0527 (4)	
C8	1.0844 (2)	0.8189 (2)	1.00708 (10)	0.0516 (3)	
H1	0.465 (3)	0.218 (2)	0.7107 (11)	0.069 (5)*	
H2	0.157 (3)	0.239 (3)	0.7840 (10)	0.070 (5)*	
H3	0.218 (2)	0.4550 (19)	0.8910 (10)	0.051 (4)*	
H5	0.518 (2)	0.6691 (19)	0.9570 (9)	0.050 (4)*	
H6	0.761 (2)	0.847 (2)	1.0152 (9)	0.053 (4)*	
H7A	0.687 (3)	1.058 (2)	0.9140 (12)	0.074 (6)*	
H7B	0.909 (3)	1.037 (2)	0.8896 (11)	0.061 (5)*	
H8B	1.176 (3)	0.821 (2)	0.9662 (10)	0.056 (4)*	
H8A	1.104 (3)	0.705 (2)	1.0311 (10)	0.059 (4)*	

### Atomic displacement parameters (Å^2^)

**Table:**

	*U*^11^	*U*^22^	*U*^33^	*U*^12^	*U*^13^	*U*^23^
S1	0.0449 (2)	0.0531 (2)	0.0512 (2)	−0.00126 (14)	0.00905 (15)	−0.00792 (15)
N1	0.0473 (6)	0.0465 (6)	0.0443 (6)	−0.0070 (5)	0.0087 (5)	−0.0088 (5)
C1	0.0617 (9)	0.0484 (8)	0.0545 (8)	−0.0023 (7)	0.0005 (7)	−0.0112 (7)
C2	0.0523 (8)	0.0552 (9)	0.0631 (9)	−0.0150 (7)	−0.0003 (7)	−0.0052 (7)
C3	0.0445 (7)	0.0532 (8)	0.0491 (7)	−0.0066 (6)	0.0077 (6)	−0.0008 (6)
C4	0.0400 (6)	0.0389 (6)	0.0393 (6)	0.0006 (5)	0.0035 (5)	0.0025 (5)
C5	0.0418 (6)	0.0428 (7)	0.0389 (6)	0.0016 (5)	0.0051 (5)	0.0000 (5)
C6	0.0450 (7)	0.0441 (7)	0.0407 (6)	−0.0041 (5)	0.0084 (5)	−0.0076 (6)
C7	0.0531 (8)	0.0492 (8)	0.0518 (8)	0.0023 (6)	−0.0091 (7)	−0.0035 (6)
C8	0.0544 (8)	0.0397 (8)	0.0584 (9)	0.0050 (6)	−0.0030 (7)	−0.0044 (6)

### Geometric parameters (Å, °)

**Table:**

S1—C1	1.7038 (16)	C5—H5	0.928 (15)
S1—C4	1.7181 (13)	C6—C8	1.518 (2)
N1—C5	1.2619 (16)	C6—C7	1.524 (2)
N1—C6	1.4616 (16)	C6—H6	0.971 (16)
C1—C2	1.347 (2)	C7—C8^i^	1.522 (2)
C1—H1	0.941 (18)	C7—H7A	0.94 (2)
C2—C3	1.410 (2)	C7—H7B	0.960 (19)
C2—H2	0.900 (18)	C8—C7^i^	1.522 (2)
C3—C4	1.3736 (18)	C8—H8B	0.954 (16)
C3—H3	0.894 (15)	C8—H8A	0.951 (17)
C4—C5	1.4492 (19)		
			
C1—S1—C4	91.62 (7)	N1—C6—C7	110.09 (11)
C5—N1—C6	117.53 (11)	C8—C6—C7	109.96 (12)
C2—C1—S1	112.26 (12)	N1—C6—H6	109.6 (9)
C2—C1—H1	129.0 (11)	C8—C6—H6	108.2 (9)
S1—C1—H1	118.7 (11)	C7—C6—H6	109.8 (9)
C1—C2—C3	112.85 (14)	C8^i^—C7—C6	111.09 (12)
C1—C2—H2	122.3 (11)	C8^i^—C7—H7A	111.4 (11)
C3—C2—H2	124.8 (11)	C6—C7—H7A	110.0 (11)
C4—C3—C2	112.27 (13)	C8^i^—C7—H7B	111.0 (11)
C4—C3—H3	122.4 (10)	C6—C7—H7B	108.7 (10)
C2—C3—H3	125.3 (10)	H7A—C7—H7B	104.5 (15)
C3—C4—C5	127.31 (12)	C6—C8—C7^i^	111.86 (12)
C3—C4—S1	110.99 (11)	C6—C8—H8B	109.9 (10)
C5—C4—S1	121.69 (9)	C7^i^—C8—H8B	106.4 (10)
N1—C5—C4	123.14 (12)	C6—C8—H8A	112.4 (10)
N1—C5—H5	121.5 (10)	C7^i^—C8—H8A	110.0 (10)
C4—C5—H5	115.3 (10)	H8B—C8—H8A	106.0 (13)
N1—C6—C8	109.16 (11)		

Symmetry codes: (i) −*x*+2, −*y*+2, −*z*+2.
